# Analysis of Factors Associated with the Risk of Suicide in a Brazilian Capital: Cross-Sectional Study

**DOI:** 10.3390/ijerph19010373

**Published:** 2021-12-30

**Authors:** Marcos Vinicius de Carvalho Mendes, Solange Laurentino dos Santos, Claudia Cristina Lima de Castro, Betise Mery Alencar Sousa Macau Furtado, Heitor Victor Veiga da Costa, Albanita Gomes da Costa de Ceballos, Pauliana Valéria Machado Galvão, Cristine Vieira do Bonfim

**Affiliations:** 1 Center for Medical Sciences, Postgraduate Program in Public Health, Federal University of Pernambuco (UFPE), Recife 50740-000, PE, Brazil; solange.lsantos@ufpe.br (S.L.d.S.); albanitagcceballos@gmail.com (A.G.d.C.d.C.); cristine.bonfim@uol.com.br (C.V.d.B.); 2 Recife Health Department (SMS), Recife 50030-903, PE, Brazil; claudiaclcastro@gmail.com; 3 Master’s Degree in Forensic Expertise, Faculty of Dentistry, University of Pernambuco (UPE), Recife 50100-010, PE, Brazil; betisemery@gmail.com; 4 Computer Center at the Federal University of Pernambuco, Recife 50740-000, PE, Brazil; hvvc.estatistica@gmail.com; 5 Faculty of Medicine, Campus of Serra Talhada, University of Pernambuco (UPE), Recife 50100-010, PE, Brazil; pauliana.galvao@upe.br; 6 Directorate of Social Research, Foundation Joaquim Nabuco, Recife 52061-540, PE, Brazil

**Keywords:** suicide, attempted, suicide, public health surveillance, record linkage

## Abstract

Suicide and suicide attempts are considered global health problems. With regard to the main causes of unnatural deaths, Brazil ranks as the eighth country with the highest absolute number of suicides. The aim of this study was to analyze the factors associated with the risk of suicide in a metropolitan city in Brazil. This was a cross-sectional study carried out in Recife, in the northeast region of Brazil. Epidemiological surveillance was conducted on the information systems regarding attempted suicide, suicide, and undetermined deaths in the period from 2007 to 2017. A record linkage between the information systems was performed. Descriptive statistics, bivariate analysis, and logistic regression were performed with an adopted a significance level of 5%. In the city of Recife, there were 4495 suicide attempts in the period, and the most frequent suicide attempts were by single females, aged between 20 and 39 years old, and who used either medication or poison to die by suicide. A total of 141 individuals died by suicide, and exogenous intoxication was the most common method. Knowing the common characteristics and associated factors of those who attempt and die by suicide is key for the development of prevention policies and intervention strategies for suicide.

## 1. Introduction

Suicide attempts and suicides are considered global health problems, and suicide is one of the main causes of unnatural deaths [1]. It is the second most frequent cause of death among young people and 17th for the general population [1,2]. Every year, it is estimated that 800,000 people die by suicide, and suicide attempts can reach a number up to 20 times higher [2,3]. Up to 42% of individuals who have attempted suicide will later retry; this recurrence is a strong predictor of suicide [4,5].

Poor mental health and the use of alcohol and/or psychoactive substances are related to a higher rate of suicide attempts using more lethal methods [6]. Other factors that are associated with people who have attempted suicide include mental disorders, being female and aged between 20 to 40 years, and easy access to poisonous substances [7,8]. The literature also reports that being male, living alone or being single, and being over 60 years old are the biggest risk factors for suicide [1,9]. Psychological disorders and suicides are cited as the main impacts on the health of social, economic, and health system determinants [10]. Therefore, actions to mitigate suicide must be taken by each country and region [6,7].

Prevention strategies involve sectors other than health, such as education, work, police, justice, religion, law, politics, and media [11]. Multisectoral environments are able to identify individuals at risk of suicide and can help to reduce suicidal events with specific practices of mental health actions in order to intervene more quickly when triggered [12]. Therapeutic follow-up, talking about the motivations that led to attempting suicide, having family support, and participating in religious help groups were seen as effective measures to reduce suicide [12,13].

According to the World Health Organization (WHO), suicide has not received adequate attention in many countries, not due to a lack of awareness that it is an important problem, but because it is considered taboo in some countries, such as Brazil [14]. Suicide must be taken seriously to reduce underreporting [2]. Urgent and emergency services register suicides and attempted suicides as incidents such as accidental poisoning, firearm injuries, and car accidents [11,15]. Survivors or family members request that the real cause not be revealed, as they believe it is a disgrace and they will be morally judged [15].

The suicide rate in Brazil varies depending on the region, from 5 to 9.9, which is still considered low by the WHO [16]. The country ranks eighth in the world in terms of the highest absolute number of suicides, and the average rate is 4.1 per 100,000 inhabitants (6.6 per 100,000 inhabitants for men and 1.8 per 100,000 inhabitants for women) [17,18]. The northeast region, in turn, presents a rate of 3.44 per 100,000 inhabitants [17]. Suicide is potentially preventable; however, preventing suicide or a suicide attempt is a complex challenge [8].

In Brazil, the National Policy for the Prevention of Self-injury and Suicide was instituted in 2019, which consists of a permanent strategy to prevent these events and aims to treat their conditioning factors [19]. This law created a national system, with states and municipalities, for the prevention of suicide and self-mutilation and a free telephone service to assist the public [19,20]. Its objectives are: promote mental health, prevent self-harm violence, control the determinants and conditioning factors of mental health, ensure access to psychosocial care, adequately address family members and those close to suicide victims to ensure proper psychosocial assistance, inform and sensitize society about the importance of self-harm and its prevention, promote intersectoral articulation for suicide prevention, in addition to promoting the notification of events, the development and improvement of methods for the collection and analysis of data on self-harm, suicide attempts, and consummated suicides [20,21,22,23]. This is a recent policy, currently being implemented by states and municipalities in Brazil [21,22,23].

The difficulties and incapacity of health services to diagnose deaths as intentional or not leads to underreporting and poor data quality [21]. The implementation of the Violence and Accident Surveillance System (VIVA) in Brazil was an important initiative to reduce external causes [22]. The monitoring of violence and mortality for epidemiological purposes is carried out by the Information System for Notifiable Diseases (SINAN) and by the Mortality Information System (SIM) of the Unified Health System (SUS) [23]. The information on those who have attempted suicide in Brazil is recorded by SINAN [24]. SINAN and SIM are components of the Health Surveillance System of the Unified Health System (SUS) [23]. Cases of exogenous intoxication became compulsory notifications in 2004, and notifications of self-inflicted violence were added in 2014 [2,24]. Suicide attempts must be registered with SINAN and suicides must be registered with SIM [2].

The study of these two health information systems, SINAN and SIM, allows for determining whether suicide attempts lead to suicide [25]. However, in Brazil’s information system, there is not a unique code that identifies whether an individual who is registered in SINAN is also present in SIM [26,27,28]. Linking the databases using a probabilistic record linkage (RL) makes it possible to combine the databases, promoting the identification of individuals present in the two databases [25]. The RL promotes the improvement of systems and information on the quality of the data, excluding duplications and including cases that would not be identified if only one information system was used [29,30].

Suicide is a complex and multicausal phenomenon, and the identification of associated factors may support preventive interventions to reduce the risk of this occurrence. Conducting research on attempts and suicides is important for their prevention and for the promotion of health, in addition to stimulating health surveillance and improving data quality. This study contributes to the knowledge of the demographic characteristics of those who attempt or die by suicide, in addition to facilitating the identification of factors associated with suicide. The aim of this study was to analyze the factors associated with suicide in a metropolitan city in Brazil.

## 2. Materials and Methods

### 2.1. Study Location and Population

This was a cross-sectional study carried out in Recife, the capital of the state of Pernambuco, in the northeast region of Brazil. The estimated population of Recife is 1,661,017, and the demographic density is approximately 7039.64 inhab/km². The municipal human development index (MHDI) is 0.772 and the Gini index is 0.68, indicating that it is a city marked by high social vulnerability and conditions of serious poverty.

The study population consisted of individuals from both genders, aged 10 years and older, residing in Recife, and who were identified as having attempted or died by suicide. The reference period of the study was from 2007 to 2017. The initial year was chosen because it was the first year with available data and the final year was the latest one with available data at the time of the study.

The SIM database includes cases of intentionally self-inflicted injuries according to the International Statistical Classification of Diseases and Related Health Problems (ICD-10—underlying cause coded between X60 and X84). Cases reported as suicide attempts were included in the study.

Suicide attempts were categorized according to the ICD-10. For suicide, ICD-10, Y10 to Y19 was used. In addition, for self-inflicted violence, intentionally self-inflicted injuries were categorized as X60–X84, and sequelae of intentionally self-inflicted injuries were categorized as Y87. Due to the underreporting of suicides, deaths by undetermined intent were analyzed according to the ICD-10, Y10–Y34.

A detailed description of the ICD-10 classifications can be found in Table S1 in the Supplementary Materials.

### 2.2. Relationship between Suicide Attempt Event Databases

A probabilistic record linkage consists of gathering information from two distinct information systems that do not have a unique identifier code between them. This was the strategy used between the databases of suicides and suicide attempts.

The relationship between the databases was analyzed using the following variables for pairing: “patient’s name”, “mother’s name”, and “birth date”. Scores of 7.0 or higher were considered to have a high degree of agreement and those with a lower score had a low degree. All records found by data linkage were selected for manual peer review. Additionally, it was also necessary to relate suicide attempts to deaths from undetermined causes to identify underreporting. The program used for record linkage was Link Plus version 2.0.

**Figure 1:**
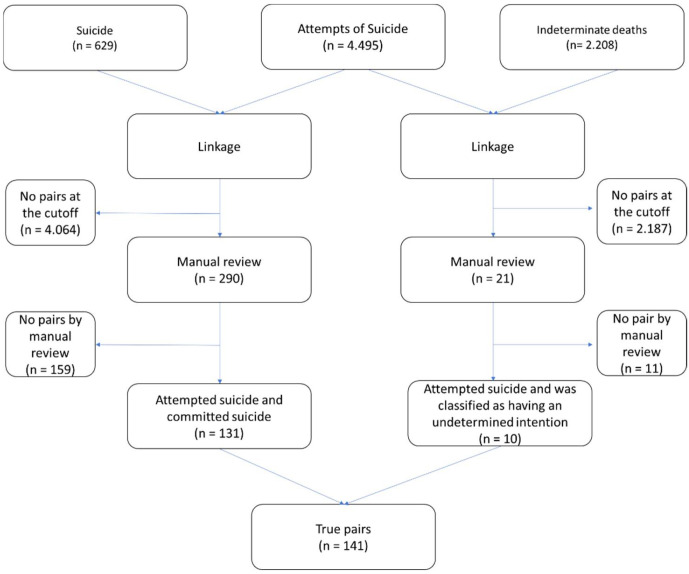
Relationship flowchart of the Information System for Notifiable Diseases (SINAN) and the Mortality Information System (SIM), Recife, from 2007 to 2017.

The analysis of the relationship between suicides and suicide attempts verified that of the 4495 attempts, 131 individuals died by suicide. It was possible to identify that 10 individuals were registered as deaths of undetermined intent. Figure 1 shows a flowchart of the relationship between the databases (Figure 1).

### 2.3. Study Variables

In the study, variables were analyzed that had completeness greater than 80%, as proposed by the Economic Commission for Latin America and the Caribbean (ECLAC) [28]. The study started with the analysis of the completeness of the variables, verifying the percentage of each variable in the study. The variables of race/color and education were not analyzed, as they had incompleteness of 24.4% and 85.6%, respectively.

The study variables were divided and categorized according to as shown below in Table 1.

### 2.4. Data Analysis

Initially, a descriptive analysis of the variables was performed using frequency, as these were categorical variables. Subsequently, bivariate analysis between the explanatory variables and the variable response (suicide) was performed. In this phase, contingency tables were constructed and the calculations of the odds ratios for the explanatory variables were performed. After bivariate analysis, the most promising explanatory variables were used to generate a multivariate logistic regression model. It was considered a promising variable when its p-value was less than or equal to 0.10.

In the modeling, crosses between the explanatory variables were considered. For the selection of model variables, the backward and forward stepwise method was adopted using the Akaike information criterion statistics (AIC) as a metric [29]. For model validation, the Hosmer and Lemeshow’s goodness-of-fit test [30] was used, with which it was identified that the model was well-adjusted if the *p*-value for this test was greater than 0.05, and the closer to 1, the better the fit.

All calculations were performed using the R programming language version 4.0.2. The significance level adopted was 5%.

### 2.5. Ethical Issues

The study was approved by the Research Ethics Committee of the Health Sciences Center of the Federal University of Pernambuco (CEP/CCS/UFPE) under CAAE number 14240919.4.0000.5208.

## 3. Results

### 3.1. Attempts Suicide

During the study period, 4495 suicide attempts were identified, with an annual average of 449. Table 2 shows the profile of suicide attempts; the majority of attempts were by females (*n* = 1344; 70.1%), aged between 20 and 39 years (*n* = 2257; 50.2%), and single (*n* = 2384; 53%). Monday was the most common day for notifications of suicide attempts (*n* = 693; 15.4%) and October was the most common month (*n* = 484; 10.8%). The region of occurrence was predominantly urban (*n* = 4123; 91.7%), and residences had the highest numbers of suicide attempts (*n*= 3431; 76.3%). The most frequent method of attempt was self-intoxication using medication (*n* = 2019; 44.9%), followed by poisoning (*n* = 1608; 35.8%). There were a number of recurrent cases (*n* = 317; 7.1%) and suicide deaths (*n* = 141; 3.1%).

### 3.2. Suicide

Of the 141 individuals who died by suicide, as shown in Table 3, the majority were male (*n* = 72; 51.1%), aged between 20 and 39 years (*n* = 68; 48.2%), and single (*n* = 81; 57.4%). Sunday and Monday were the days with the most suicides (*n* = 22; 15.6%), and July and October also had the same number of notifications (*n* = 18; 12.8%). Urban areas (*n* = 128; 90.8%) and residences were the most common locations for suicide (*n* = 93; 66.0%). Exogenous self-intoxication due to the use of poisonous products was the most commonly used method (*n* = 93; 66.0%), and death by suicide affected individuals who had made a previous attempt (*n* = 15; 10.6%).

The results of the bivariate analysis are shown in Table 4, presenting the factors associated with suicide. In terms of gender, the chance of dying by suicide was 2.53 times (*p* < 0.001) higher for males than for females. The results also show that there was a greater chance of suicide depending on the age. Taking the age group of 10 to 19 years as the baseline, the chances are higher in 2.66 (*p* < 0.001) for individuals aged 20 to 39 years, 4.57 (*p* < 0.001) for individuals aged 40 to 59 years, and 7.26 (*p* < 0.001) for individuals aged 60 years and over. Those who used medication or cosmetic or hygiene products had lower chances of 75% and 85%, respectively, while for those who poisoned themselves, the chances of suicide increased by 4.24 times (*p* < 0.001). Hanging also appeared as a method, with the chances of suicide 4.26 times higher (*p* < 0.04) than those by other methods. The chances of dying by suicide were 1.71 times higher (*p* < 0.01) in public places compared to attempts at home. The variables of recidivism and marital status presented *p*-values to be included in the multivariate analysis (*p* < 0.10).

The Hosmer and Lemeshow test obtained a *p*-value of 0.59, showing that the model has a good goodness-of-fit. The results are shown in Table 5 with the multivariate logistic regression model. The chances of single individuals dying by suicide were 1.79 higher compared to those who are married/in a stable relationship (*p* < 0.04). Men had 1.85 times the chance of dying by suicide compared to women (*p* < 0.001). The effect of the age group variable strictly increased, with the chances of dying by suicide 2.33 (*p* = 0.02), 4.19 (*p* < 0.001), and 5.6 (*p* < 0.001) times higher for the age groups from 20 to 39, 40 to 60, and over 60 years, respectively, than the age group from 10 to 19 years. Attempting suicide by consuming poisonous products was 4.59 times (*p* < 0.01) more likely to result in death by suicide than other methods. Repeated attempts were 2.19 times more likely to result in death by suicide compared to those who tried for the first time (*p* < 0.01).

## 4. Discussion

The results show that most suicide attempts were by single females, aged between 20 and 39 years old, and who used poison as a method, as has already been found in previous studies [16,31,32]. The factors associated with suicide death were the male gender, the age group of 40 years or more, and recurrent attempts. The results indicate there was a relationship between the age group and gender, but the estimates changed during the application of the model. This suggests that the effect of the age group on the rate of suicide changed with the gender by age group.

These results are in agreement with the literature [33,34]. A study using Slovenian suicide records showed that drug use and a mean age of 30 years were linked to attempted suicide in females [35]. When women die of suicide, it is mostly through the use of pesticides [16]. Furthermore, one study noted that the use of alcohol also facilitates attempts, as it has a potential factor in increasing dysphoria and accentuating anxieties or other disorders [36].

The study showed that male individuals were more likely to attempt suicide and die from it, and that they most often used poisoning as the method. A reduction in the suicide rate can be achieved through stricter legislation on access to poisonous materials, such as pesticides [6]. It has been seen that countries with a stricter pesticide purchasing policy have reduced the number of suicides [37,38]. It is possible to understand that suicide can be related to the culture and accessibility to the suicide method in each country and region [39,40].

Poisoning had a greater chance of causing suicide, while the use of medication and consumption of hygiene products and cosmetics had lower chances. The means to resolve a situation of high consumption of medication tablets or liquid products is through a stomach wash, which is available at all levels of care [2]. Thus, decreasing an individual’s risk of death by this method has greater support and better success rates [34].

It was also observed in the study that women attempt more suicides than men, but they generally fail to achieve it. The literature shows that this is due to the use of more violent methods such as jumping from great heights, hanging, and firearms are the most common choices for suicide among men [22,23,24]. In countries that have a policy of releasing firearms, cases of suicide by this means occur more frequently [22]. A reduction in the suicide rate can be made from stricter legislation regarding the most commonly used methods in each region [24].

All age groups were related to suicide attempts. Adolescents aged between 15 and 19 years were more likely to engage in more aggressive behavior, consume more alcohol, and become endangered [41]. For the young adult population, their risk of suicide may have been associated with separation or the loss of a spouse, unemployment—with which the risk of suicide becomes greater in times of crisis—and work incapacity, which can lead to other psychological and psychiatric disorders [16,42,43]. Elderly people, in turn, are afraid of becoming totally dependent and are prone to developing depression. In addition, other diseases of old age—such as cancer, diabetes, and hypertension—can increase the need for greater care, which generates discontent among the elderly, predicting a suicide attempt or suicide death [44,45,46].

Multivariate analysis showed recurrent attempts as an associated factor of suicide. The results of this study support the evidence that previous suicide attempt is the crucial factor for consummated suicide [3,5,47,48]. A study using applied logistic regression noted that a family history of suicide, mental disorder, hopelessness, and a lower level of social support were significant predictors of multiple suicide attempts [5]. Studies have highlighted that nearly 20% of individuals with a previous suicide attempt recidivate primarily within the first month [3,49]. For those who have reported a suicide attempt, intensive monitoring during the critical period is paramount to prevent a secondary attempt.

In some studies, individuals who have attempted suicide more than once were more likely to have problems related to mental and physical health when, generally, they reached middle age [1,50]. People with a disease such as cancer and/or an unfavorable financial situation are more likely to have recurrent attempts compared to the general population [43,51]. Countries that do not have a suicide intervention or prevention policy may have higher numbers of suicide attempts [15]. Brazil has a volunteer organization known as the Life Valorization Center (CVV) which provides the phone number “188” to individuals in need of support [52,53].

Being single had a significant influence on suicide deaths after a previous suicide attempt. Social connections are considered protective factors against suicide, as already described in the literature [14,43,54,55]. Furthermore, isolation, greater consumption of alcohol and other substances, and financial difficulties can accentuate social isolation, predisposing a suicide attempt [14,43]. Therefore, the creation of friendship circles and participating in support groups can help to reduce suicide rates [56]. For Turecki et al. (2019) [57], prevention is essential to reduce the number of suicides, and involves a combination of universal, selective, and specific strategies, including psychological and pharmacological treatments and social and public health initiatives.

Public places were associated with suicides in this study. Spaces with few people or abandoned buildings, as well as buildings of great heights are listed in the literature as spaces with greater risk [39]. Installing support points, creating access barriers, and encouraging the search for help on posters or visual media can prevent some suicides and better educate the population on the subject [58].

Underreporting was verified in this study through record linkage, with suicides reported as deaths of undetermined intent. Non-notification of suicides occurs when the individual is taken to the emergency room of a health service that denies a suicide attempt [8]. Failures to disclose the basic cause of death by the coroner may also occur [19,20].

### 4.1. Strong Points and Limitations

One of the strengths of the study was the record linkage, which made it possible to link multiple information systems and retrieve underreported data. The linkage record made it possible to correlate the data of 10 years of suicide attempts and suicides, and to identify individuals who attempted suicide and who died. This practice is effective and can be routinely used in health services to reduce underreporting and identify individuals in different information systems [25]. Another highlight is the sample size of people who have attempted suicide, which made it possible to obtain relevant information about the risk of new attempts. This information can be useful for planning suicide prevention measures.

A limiting factor of the study is that secondary data were used. The incompleteness of the information and the underreporting prevented the identification of suicides. FAn analysis of the completeness of the variables was performed and only those with good completeness remained in the study. The variables education and race/color, which can be used as proxy for social inequality, could not be analyzed. The suicide attempts analyzed came from the health services, which means that those that did not require care in health units may have been lost. Therefore, it is possible that the notifications are of more clinically serious cases. There may also be difficulties for medical examiners to identify causes of death as suicides, considering a possible underreporting of suicides. The study identified individuals whose underlying cause of death was an event of undetermined intent, but linkage of the databases allowed the location of a previous record of attempted suicide.

Information on income, access to services, and mental health was not available in the analyzed databases. Typing errors in the data of individuals can cause difficulties in the identification of true pairs by probabilistic record linkage.

The results of this study can contribute to the National Policy for the Prevention of Self-injury and Suicide, in terms of its goals of prevention and identification of factors associated with suicides.

### 4.2. Implications and Directions for Future Research

The study brings some implications to be addressed in suicide prevention. Some factors associated with suicide identified by this study were the male gender, an age group of 40 years or older, and the recidivism of a suicide attempt. This information is relevant for suicide prevention programs to be tailored to the specific profiles of each group, which should be monitored by mental health services and primary health care.

There is a need for a greater offer of psychological follow-up by health services, support for pharmacological treatment when necessary and indicated by a medical specialist, and studies to evaluate the health surveillance of self-inflicted violence. Studies that relate diverse databases and have information about suicide attempts, psychiatric disorders, and substance use should be conducted, aiming at surveillance and prevention.

The relationship of databases of suicide attempts and suicides should be applied in the routine of health services, to identify individuals who need immediate attention to ensure psychosocial care. Reducing the risk of new suicide attempts is possible, and telephone intervention programs can be considered an example. In the study conducted by Parra-Urribe et al. (2017) [49], all first suicide attempts who were discharged from the emergency room had an appointment with the psychiatrist scheduled for a maximum period of 10 days and consented to participate in a telephone follow-up program for one year. This could be a strategy adopted by the health services.

There is a need to encourage the improvement of official records, especially regarding attempts. Improving the reporting of suicide attempts and suicides is one of the goals of the National Policy for the Prevention of Self-injury and Suicide, and the improvement of information is important to support policy formulation and decision making.

In addition, integrating multisectoral policies on access to some methods for suicide attempts, such as greater control on the purchase of pesticides, high-control/black market medications, and the difficulty of access to carry a firearm, as well as security measures in high places, will be effective in preventing suicide.

## 5. Conclusions

Studying the suicide attempt databases and relating them to suicides enabled the identification of the profile of individuals and associated factors. Female gender, age between 20 and 40 years, and the use of exogenous self-intoxication were the main characteristics of this population that attempted suicide. Factors associated with suicide are male gender, age of 40 years or more, and recurrent attempts. The strengthening of monitoring and surveillance actions for these occurrences must be implemented as a way to protect human health, life, and well-being. Improving health information systems and the knowledge and awareness of professionals about the intrinsic factors in the psychosocial and environmental dimensions involved in the determination must be a priority for managers and decision makers.

## Figures and Tables

**Table 1:** Description of the variables used and analyzed in the study.

Variables	Categorization
Sociodemographic characteristics	
Gender	Female
	Male
Age group in years	10 to 19
	20 to 39
	50 to 60
	Over 60
Marital Status	Married/stable union
	Single
	Widow
Day, month, and geographic location of occurrence of suicide attempt or suicide	
Day of the week of occurrence	Sunday
	Monday
	Tuesday
	Friday
	Saturday
Month of occurrence	January
	February
	March
	April
	May
	June
	July
	August
	September
	October
	November
	December
Place of occurrence	Urban
	Rural
Place of occurrence	Residence
	Collective housing
	School
	Sports practice venue
	Bar or similar
	Public highway
	Commerce/services
	Other
Method of suicide attempt or suicide	
Method of suicide attempt	Medication
	Poisoning
	Cosmetic or hygiene products
	Body strength
	Hanging
	Blunt object
	Sharp object
	Hot substance or object
	Firearm
	Suicide crisis/outbreak
Method of suicide	Medication
	Poisoning
	Cosmetic and hygiene products
	Body strength
	Hanging
	Sharp object
	Hot substance or object
Outcome of suicide attempt	
Recurrent (a)	No
	Yes
Suicide	No
	Yes

(a) Repeated suicide attempts are considered a risk factor for suicide and in this study.

**Table 2:** Distribution of demographic characteristics according to day, month, location, and method of suicide attempts in Recife, Pernambuco, Brazil, 2007–2017.

Variable	n = 4495	%
Sociodemographic characteristics		
Gender (a)		
Female	3150	70.1
Male	1344	29.9
Age group in years		
10 to 19	1128	25.1
20 to 39	2257	50.2
40 to 60	969	21.6
Over 60	141	3.1
Marital status (b)		
Married/stable union	928	20.6
Single	2384	53.0
Widow	495	11.0
Day, month, and geographic location		
Day of the week of occurrence		
Sunday	675	15.0
Monday	693	15.4
Tuesday	637	14.2
Wednesday	619	13.8
Thursday	633	14.1
Friday	604	13.4
Saturday	634	14.1
Month of occurrence		
January	373	8.3
February	335	7.5
March	322	7.2
April	353	7.9
May	347	7.7
June	342	7.6
July	367	8.2
August	409	9.1
September	427	9.5
October	484	10.8
November	375	8.3
December	361	8.0
Place of occurrence (c)		
Urban	4123	91.7
Rural	5	0.1
Place of occurrence (d)		
Residence	3431	76.3
Collective housing	18	0.4
School	6	0.1
Sports practice venue	7	0.2
Bar or similar	9	0.2
Public highway	99	2.2
Commerce/services	13	0.3
Other	704	15.7
Method of suicide attempt §		
Medication	2019	44.9
Poisoning	1608	35.8
Cosmetic or hygiene products	190	4.2
Body strength	122	2.7
Hanging	25	0.6
Blunt object	24	0.5
Sharp object	98	2.2
Hot substance or object	26	0.6
Firearm	26	0.6
Suicide crisis/outbreak	42	0.9
Outcome of suicide attempt		
Recurrent		
No	4178	92.9
Yes	317	7.1
Suicide		
No	4354	96.9
Yes	141	3.1

Missing data: (a) 0.02% (*n* = 1); (b) 15.3% (*n* = 688); (c) 8.1% (*n* = 367); (d) 4.6% (*n* = 208). § Variable of multiple responses, so the sum of their relative frequencies can be greater than 100%.

**Table 3:** Distribution of demographic characteristics according to day, month, location, and underlying cause of suicides in Recife, Pernambuco, Brazil, 2007–2017.

Variable	n = 141	%
Sociodemographic characteristics		
Gender		
Female	69	48.9
Male	72	51.1
Age group in years		
10 to 19	13	9.2
20 to 39	68	48.2
40 to 60	49	34.8
Over 60	11	7.8
Marital status (a)		
Married/stable union	20	14.2
Single	81	57.4
Widow	14	9.9
Day, month, and geographic location		
Day of the week of occurrence		
Sunday	22	15.6
Monday	22	15.6
Tuesday	13	9.2
Wednesday	28	19.9
Thursday	19	13.5
Friday	24	17.0
Saturday	13	9.2
Month of occurrence		
January	11	7.8
February	17	12.1
March	4	2.8
April	8	5.7
May	14	9.9
June	8	5.7
July	18	12.8
August	13	9.2
September	11	7.8
October	18	12.8
November	7	5.0
December	12	8.5
Place of occurrence (b)		
Urban	128	90.8
Place of occurrence (c)		
Residence	93	66.0
Collective housing	1	0.7
Public highway	5	3.5
Other	33	23.4
Method of suicide		
Medication	24	17.0
Poisoning	93	66.0
Cosmetic and hygiene products	1	0.7
Body strength	1	0.7
Hanging	3	2.1
Sharp object	2	1.4
Hot substance or object	2	1.4

Missing data: (a) 18.4% (*n* = 26); (b) 9.2% (*n* = 13); (c) 6.4% (*n* = 9). Multiple-response variable, so the sum of their relative frequencies can be greater than 100%.

**Table 4:** Factors associated with demographic characteristics of suicides in Recife, Pernambuco, Brazil, 2007–2017.

Variable	Suicide				Odds Ratio			
	No (n = 4354)		Yes (n = 141)					
	n	%	n	%	Estimation	IC 95%		*p*-Value
Sociodemographic characteristics								
Gender								
Female	3081	70.8	69	48.9	1.00	-	-	-
Male	1272	29.2	72	51.1	2.53	1.81	3.54	<0.001
Age								
10 to 19	1115	25.6	13	9.2	1.00	-	-	-
20 to 39	2189	50.3	68	48.2	2.66	1.47	4.84	<0.001
40 to 60	920	21.1	49	34.8	4.57	2.46	8.47	<0.001
Over 60	130	3.0	11	7.8	7.26	3.19	16.53	<0.001
Marital status								
Married/stable union	908	20.9	20	14.2	1.00	-	-	-
Single	2303	52.9	81	57.4	1.60	0.97	2.62	0.07
Widow	481	11.0	14	9.9	1.32	0.66	2.64	0.47
Day, month, and geographic location of suicide attempts								
Day of the week of occurrence								
Sunday	653	15.0	22	15.6	1.00	-	-	-
Monday	671	15.4	22	15.6	0.97	0.53	1.77	1.00
Tuesday	624	14.3	13	9.2	0.62	0.31	1.24	0.23
Wednesday	591	13.6	28	19.9	1.41	0.80	2.49	0.25
Thursday	614	14.1	19	13.5	0.92	0.49	1.71	0.87
Friday	580	13.3	24	17.0	1.23	0.68	2.21	0.55
Saturday	621	14.3	13	9.2	0.62	0.31	1.24	0.23
Month of occurrence								
January	362	8.3	11	7.8	1.00	-	-	-
February	318	7.3	17	12.1	1.76	0.81	3.81	0.18
March	318	7.3	4	2.8	0.41	0.13	1.31	0.19
April	345	7.9	8	5.7	0.76	0.30	1.92	0.65
May	333	7.6	14	9.9	1.38	0.62	3.09	0.54
June	334	7.7	8	5.7	0.79	0.31	1.98	0.65
July	349	8.0	18	12.8	1.70	0.79	3.65	0.19
August	396	9.1	13	9.2	1.08	0.48	2.44	1.00
September	416	9.6	11	7.8	0.87	0.37	2.03	0.83
October	466	10.7	18	12.8	1.27	0.59	2.73	0.57
November	368	8.5	7	5.0	0.63	0.24	1.63	0.35
December	349	8.0	12	8.5	1.13	0.49	2.60	0.83
Occurrence zone								
Urban	3995	91.8	128	90.8	1.00	-	-	-
Countryside/Periurban	5	0.1	0	0.0	0.00	-	-	-
Place of occurrence								
Residence	3338	76.7	93	66.0	1.00	-	-	-
Public place	817	18.8	39	27.7	1.71	1.17	2.51	0.01
Suicide method								
Medication								
Yes	2280	52.4	110	78.0	1.00	-	-	-
No	1995	45.8	24	17.0	0.25	0.16	0.39	<0.001
Poisoning								
Yes	2622	60.2	38	27.0	1.00	-	-	-
No	1515	34.8	93	66.0	4.24	2.89	6.21	<0.001
Cosmetic and hygiene products								
Yes	4086	93.8	133	94.3	1.00	-	-	-
No	189	4.3	1	0.7	0.16	0.02	1.17	0.03
Body strength								
Yes	4077	93.6	133	94.3	1.00	-	-	-
No	121	2.8	1	0.7	0.25	0.04	1.83	0.18
Hanging								
Yes	4142	95.2	132	93.6	1.00	-	-	-
No	22	0.5	3	2.1	4.28	1.27	14.48	0.04
Blunt object								
Yes	4142	95.1	134	95.0	1.00	-	-	-
No	24	0.6	0	0.0	0.00	-	-	-
Cutting object								
Yes	4096	94.1	132	93.6	1.00	-	-	-
No	96	2.2	2	1.4	0.65	0.16	2.65	0.77
Hot substance or object								
Yes	4141	95.1	133	94.3	1.00	-	-	-
No	24	0.6	2	1.4	2.59	0.61	11.09	0.20
Fire gun								
Yes	4145	95.2	133	94.3	1.00	-	-	-
No	26	0.6	0	0.0	0.00	-	-	-
Suicide crisis/outbreak								
Yes	4119	94.6	133	94.3	1.00	-	-	-
No	42	1.0	0	0.0	0.00	-	-	-
Attempted suicide more than once								
Recurrent								
No	4052	93.1	126	89.4	1.00	-	-	-
Yes	302	6.9	15	10.6	1.60	0.92	2.76	0.09

**Table 5:** Results of the multivariate logistic regression model.

Coefficients	Odds Ratio			
	Estimation	IC 95%		*p*-Value
Intercept	-	-	-	<0.001
Age group (20 to 39)	2.33	1.18	5.13	0.02
Age group (40 to 60)	4.19	2.07	9.39	<0.001
Age group (Over 60)	5.60	1.91	15.80	<0.001
Gender (Male)	1.85	1.23	2.79	<0.001
Suicidal Method—Poisoning (Yes)	4.59	1.16	3.87	0.01
Marital status (Single)	1.79	1.06	3.18	0.04
Marital status (Widow)	1.43	0.67	3.01	0.34
Place of occurrence (Public place)	1.52	0.94	2.38	0.08
Recurrent (Yes)	2.19	1.16	3.87	0.01

## Data Availability

The data are available by contacting the corresponding author.

## Supplementary Materials

The following are available online at https://www.mdpi.com/article/10.3390/ijerph19010373/s1. Table S1: Description of suicide attempt, suicide and deaths by undetermined in-tent according to the International Statistical Classification of Diseases and Relat-ed Health Problems (ICD-10).
