# Supplementary material for: Analysis of Factors Associated with the Risk of Suicide in a Brazilian Capital: Cross-Sectional Study
Source: Int J Environ Res Public Health. 2021 Dec 30;19(1):373. doi: 10.3390/ijerph19010373 (PMC8751138; doi:10.3390/ijerph19010373)
Supplement: Supplementary file 1 [file ijerph-19-00373-s001.zip › ijerph-1495721-supplementary.pdf]

International Journal of
Environmental Research and Public Health

MDPI

Article – IJERPH - 1495721

Supplementary Materials

Tabela S1: Description of suicide attempt, suicide and deaths by undetermined intent according to the International Statistical Classification of Diseases and Related Health Problems (ICD-10).

ICD-10	Description
Suicide attempt and suicide	
X60	Intentional self-poisoning by and exposure to nonopioid analgesics, antipyretics and antirheumatics
X61	Intentional self-poisoning by and exposure to antiepileptic, sedative-hypnotic, antiparkinsonism and psychotropic drugs, not elsewhere classified
X62	Intentional self-poisoning by and exposure to narcotics and psychodysleptics [hallucinogens], not elsewhere classified
X63	Intentional self-poisoning by and exposure to other drugs acting on the autonomic nervous system
X64	Intentional self-poisoning by and exposure to other and unspecified drugs, medicaments and biological substances
X65	Intentional self-poisoning by and exposure to alcohol
X66	Intentional self-poisoning by and exposure to organic solvents and halogenated hydrocarbons and their vapours
X67	Intentional self-poisoning by and exposure to carbon monoxide and other gases and vapours
X68	Intentional self-poisoning by and exposure to pesticides
X69	Intentional self-poisoning by and exposure to other and unspecified chemicals and noxious substances
X70	Intentional self-harm by hanging, strangulation and suffocation
X71	Intentional self-harm by drowning and submersion
X72	Intentional self-harm by handgun discharge
X73	Intentional self-harm by rifle, shotgun and larger firearm discharge
X74	Intentional self-harm by other and unspecified firearm discharge
X75	Intentional self-harm by explosive material
X76	Intentional self-harm by smoke, fire and flames
X77	Intentional self-harm by steam, hot vapours and hot objects
X78	Intentional self-harm by sharp object
X79	Intentional self-harm by blunt object
X80	Intentional self-harm by jumping from a high place
X81	Intentional self-harm by jumping or lying before moving object
X82	Intentional self-harm by crashing of motor vehicle
X83	Intentional self-harm by other specified means
X84	Intentional self-harm by unspecified means
Deaths by undetermined intent	
Y10	Poisoning by and exposure to nonopioid analgesics, antipyretics and antirheumatics, undetermined intent
Y11	Poisoning by and exposure to antiepileptic, sedative-hypnotic, antiparkinsonism and psychotropic drugs, not elsewhere classified, undetermined intent
Y12	Poisoning by and exposure to narcotics and psychodysleptics [hallucinogens], not elsewhere classified, undetermined intent
Y13	Poisoning by and exposure to other drugs acting on the autonomic nervous system, undetermined intent
Y14	Poisoning by and exposure to other and unspecified drugs, medicaments and

	biological substances, undetermined intent
Y15	Poisoning by and exposure to alcohol, undetermined intent
Y16	Poisoning by and exposure to organic solvents and halogenated hydrocarbons and their vapours, undetermined intent
Y17	Poisoning by and exposure to carbon monoxide and other gases and vapours, undetermined intent
Y18	Poisoning by and exposure to pesticides, undetermined intent
Y19	Poisoning by and exposure to other and unspecified chemicals and noxious substances, undetermined intent
Y20	Hanging, strangulation and suffocation, undetermined intent
Y21	Drowning and submersion, undetermined intent
Y22	Handgun discharge, undetermined intent
Y23	Rifle, shotgun and larger firearm discharge, undetermined intent
Y24	Other and unspecified firearm discharge, undetermined intent
Y25	Contact with explosive material, undetermined intent
Y26	Exposure to smoke, fire and flames, undetermined intent
Y27	Contact with steam, hot vapours and hot objects, undetermined intent
Y28	Contact with sharp object, undetermined intent
Y29	Contact with blunt object, undetermined intent
Y30	Falling, jumping or pushed from a high place, undetermined intent
Y31	Falling, lying or running before or into moving object, undetermined intent
Y32	Crashing of motor vehicle, undetermined intent
Y33	Other specified events, undetermined intent
Y34	Unspecified event, undetermined intent
